# Supramolecular electrode assemblies for bioelectrochemistry

**DOI:** 10.1039/c7cc01154g

**Published:** 2017-03-20

**Authors:** Theodoros Laftsoglou, Lars J. C. Jeuken

**Affiliations:** a School of Biomedical Sciences and Astbury Centre for Structural Molecular Biology , University of Leeds , LS2 9JT , Leeds , UK . Email: l.j.c.jeuken@leeds.ac.uk

## Abstract

For more than three decades, the field of bioelectrochemistry has provided novel insights into the catalytic mechanisms of enzymes, the principles that govern biological electron transfer, and has elucidated the basic principles for bioelectrocatalytic systems. Progress in biochemistry, bionanotechnology, and our ever increasing ability to control the chemistry and structure of electrode surfaces has enabled the study of ever more complex systems with bioelectrochemistry. This feature article highlights developments over the last decade, where supramolecular approaches have been employed to develop electrode assemblies that increase enzyme loading on the electrode or create more biocompatible environments for membrane enzymes. Two approaches are particularly highlighted: the use of layer-by-layer assembly, and the modification of electrodes with planar lipid membranes.

## Abstract 2

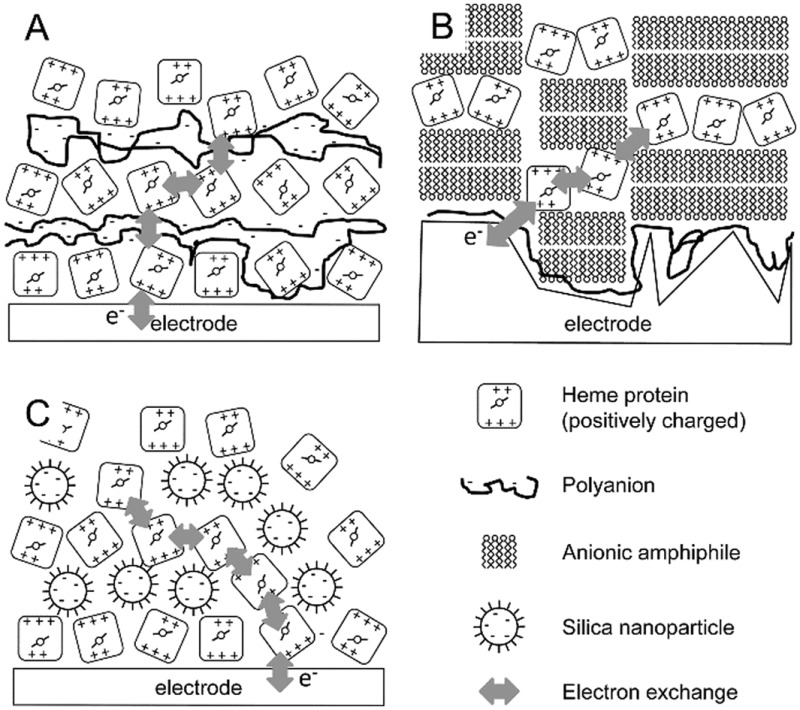
Supramolecular approaches in bioelectrochemistry have boosted enzyme loading on electrodes and shaped biocompatible environments for enzymes.

## Introduction

Redox active proteins are ubiquitous in nature and are involved in a plethora of metabolic processes, where they mediate electron transport and catalyse many metabolic and industrially important redox reactions. Proteins that function solely in electron transport contain typically a single domain and are less than 40 kDa in size. In contrast, many redox enzymes are large multi-subunit complexes and a significant number of those active in photosynthesis and respiration are integral membrane proteins. Our understanding of protein electron transport, along with the characterisation of redox active enzymes has been aided by the electrochemical investigation of these enzymes. A very powerful electrochemical technique, which was developed in the eighties, has been protein film electrochemistry (PFE), where a protein film is formed on an electrode surface such that electrons flow directly between the electrode and the adsorbed redox active proteins.^1–4^ PFE electrochemistry was initially performed with pyrolytic graphite ‘edge’ (PGE) electrodes that provide a very heterogeneous negatively-charged surface on which proteins adsorb without significant disturbance to their native fold and, if required, the surface charge could be tailored with polycations such as polymixin B.

In addition, electrodes were modified with biomembrane-like films^5,6^ or self-assembled monolayers (typically on gold electrodes),^7^ while the proteins themselves could also be engineered to optimise the formation of a film.^8^ Finally, electrochemical techniques were extended by sensitive techniques like scanning tunnelling microscopy.^9^

Although PFE has since its conception been proven invaluable towards our understanding of a plethora of metalloproteins, it does not come without limitations for bioelectrocatalytic and other biotechnological applications. In particular, being a surface-based technique, there are the obvious limitations in the amount of enzyme that can be adsorbed on the electrode's surface, while mass transport can limit access to substrate. To enhance the enzyme ‘loading’ on an electrode, procedures have emerged to form mesoporous,^10^ ‘3D’ electrodes^11^ or electrode architectures that consist of protein multilayers.^12^ Another limitation of PFE is encountered with certain complex multi-subunit enzymes and, in particular, membrane enzymes, where interfacial electron transfer might be limiting and different surface chemistries are needed in order to orientate or ‘wire’ those enzymes to the electrode without perturbing their native tertiary or quaternary folds. Examples were surface chemistry has been optimised to control enzyme orientation and electrocatalysis include laccase^13,14^ and bilirubin oxidase^15^ for oxygen reduction and photosystem II (PSII) for water oxidation.^16^

In this feature article, we look at recent examples where supramolecular electrode assemblies have provided solutions for the limitations addressed above. Supramolecular chemistry is concerned with systems that are comprised of molecular units that are assembled by weak interactions; they are primarily focused on electrostatic, van der Waals and hydrophobic interactions, and, more recently, metal coordination chemistry. Although supramolecular chemistry underpins all approaches of electrode assemblies, this feature will focus on two recent approaches. First, we will discuss recent progress in the formation of multilayer or multicomponent complex electrode assemblies where the redox proteins retain efficient electron exchange with the electrode. Second, we will discuss how supramolecular electrode assemblies have been used to accommodate and exploit integral membrane enzymes in bioelectrochemistry.

## Multilayer protein assemblies

An early pioneering approach towards multilayer protein assemblies arrived from the Rusling group, where the surfactant didodecyldimethylammonium bromide (DDAB), polyanions or clays were employed for the formation of stable thin films onto PG electrodes that support the spontaneous incorporation of heme proteins such as myoglobin or hemoglobin.^6,17–21^ These DDAB films displayed enhanced redox signals and catalysed a number of redox reactions such as NO reduction and dehalogenation of organohalides.^6,22^ Although catalysis in DDAB was argued by the Koper group to be due to free heme, released by protein denaturation,^23,24^ further spectroscopic and electrochemical evidence by the Rusling group has shown that the majority of myoglobin is in its native fold.^25^

When redox proteins in thin films are not mobile enough to diffuse to and from the electrode, redox active compounds, can be employed to mediate electron transfer. For example, osmium polypyridyl complex, sodium dodecyl sulfate (SDS), and glucose oxidase were used to form a multicomposite multilayer assembly that was driven by hydrophobic as well as electrostatic interactions.^26^ Cyclic voltammetry showed a linear increase of the redox signals up to 4 ‘dipping cycles’, while a 3-fold increase in glucose oxidase coverage was seen with SDS that led to a 9-fold increase in sensitivity for the detection of glucose at a sensitivity of 18.4 μA cm^–2^ per layer.^26^

As small redox compounds tend to leak out of thin film assemblies, attaching the mediators either covalently or *via* coordinate bonds will enhance the stability of the system. As the mediators are not free to diffuse in these systems, high enough concentrations need to be used to ensure efficient electron hopping between the mediators. Systems employing bound mediators, in particular osmium complexes and methyl viologen, were first described by Heller^27^ and later optimised by, among others, Schuhmann and Plumeré.^28–30^ In a recent study, a naphthoquinone was also used to create redox hydrogel, which increased maximum current density and maximum power density in fuel cell based on glucose dehydrogenase.^31^

### Layer-by-layer assembly

**Fig. 1:**
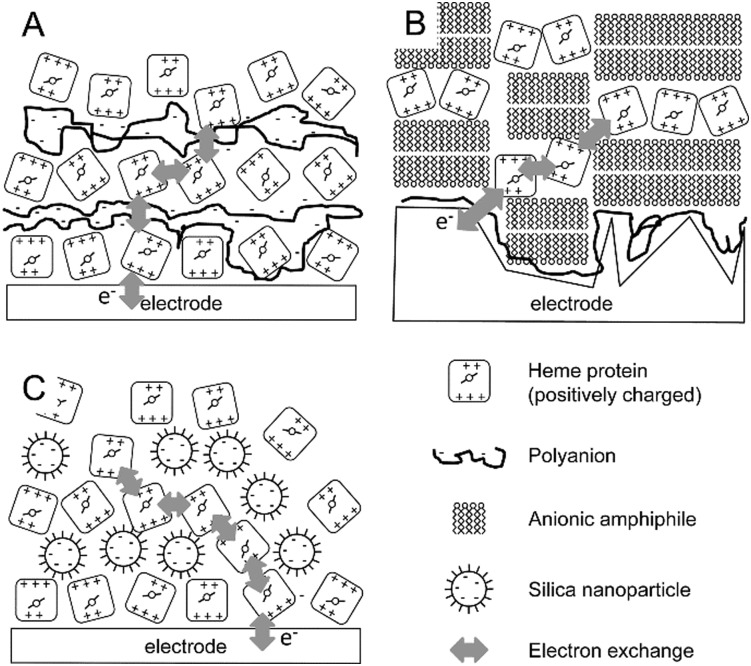
Schematic representation of three layer-by-layer assemblies in bioelectrochemistry. (A) Layer-by-layer assemblies of polyanions and a positively charged redox protein. (B) Layer-by-layer assemblies of a cationic amphiphile forming bilayers and positively charged redox proteins. (C) Layer-by-layer assemblies of negatively charged nanoparticles and positively charged redox proteins. Although examples are given for positively charged redox proteins (*i.e.*, systems where the pH < pI of the protein), similar assemblies can be proposed where the charges are reversed.

In contrast to trapping proteins in thin films, the more controlled process of layer-by-layer assembly has been exploited in generating protein multilayers for bioelectrochemical applications. For instance, poly(methacrylic acid) (PMAA) was used to form multilayers of myoglobin that were electronically coupled (Fig. 1A), as shown by the increasing electroactive myoglobin up to 13 layers during cyclic voltammetry reaching just over 150 pmol cm^–2^ of protein.^32^ Moreover, it was shown that the amount of electroactive myoglobin is pH dependent (pH 5.0 > pH 6.5 > pH 8.0).^32^

In a similar approach, layer-by-layer assemblies of the anionic surfactant dihexadecyl phosphate (DHP) have been formed, intercalated with positively charged myoglobin or haemoglobin (Fig. 1B).^33^ The amphiphilic nature of DHP induces self-assembly into planar bilayers on the electrode surface. Layer-by-layer assembly was monitored electrochemically and redox signals from the haemoglobin increased linearly in magnitude up to 6 layers. The total protein content was also shown to increase linearly by UV-visual spectroscopy.^33^ This system was demonstrated to be stable up to 2 weeks of storage in buffer with pH 7.0. The authors proposed that electron transfer to the heme proteins in the layered system proceeds *via* a hopping mechanism. Importantly, electrochemistry was obtained on roughened pyrolytic graphite electrodes (equivalent to PGE) and this could be important as geometrically perfect DHP bilayers would block electron transfer between heme proteins on either side of the DHP bilayers.

It must be noted that these types of layer-by-layer multiprotein assemblies primarily focus on hemoglobin^34,35^ and myoglobin.^36^ Both proteins have non-covalent redox-active cofactors (heme b) that could potentially be released from small amounts of denatured protein and give rise to electroactive signals and, therefore, such methods need to be approached with due care.

An alternative, but equally easy to implement approach to create protein multilayers has been reported by the group of Lisdat, where the formation of cytochrome *c* protein multilayers can be supported by modified silica nanoparticles (Fig. 1C).^37,38^ Layers of cytochrome *c* and of the silica nanoparticles were formed by alternate incubations of each component, where the assembly was guided and stabilized by electrostatic interactions. It was shown that electronic coupling, by electron hopping between the cytochrome *c* molecules, can be achieved for up to 5 protein layers with a linearly increasing amount of electroactive cytochrome *c* from ∼8 to ∼80 pmol cm^–2^.^37^ The importance of the silica nanoparticle size was also demonstrated. Small (5–15 nm diameter) nanoparticles showed successful layer formation, while no layer formation was observed by quartz-crystal microbalance (QCM) studies for larger silica particles (20–60 nm diameter).^37^

### Layer-by-layer assembly using conducting layers

So far, examples have been described in which redox-active proteins have been deposited using non-conducting ‘adhesive’ interlayers and hence electron transfer relies on electron hopping between the redox proteins in the different layers. However, it should be possible to use conductive or redox-active adhesive layers, for instance gold nanoparticles. The benefits of gold nanoparticles, and by extension metal nanoparticles, in bioelectrochemistry have been described more than a decade ago. They do not only increase the surface area, but also mediate electron transfer. In one of the first examples of this kind, apo-glucose oxidase was reconstituted with gold nanoparticles baring the enzyme's native cofactor, FAD, both *in vitro* and *in situ*.^39^ A 7-fold increase in the unimolecular electron transfer rate was observed, demonstrating that gold nanoparticles indeed increase the maximum turn-over rate.^39^

The potential of gold nanoparticles in bioelectrochemistry was more recently shown when gold nanoparticles were modified with alkyl thiols and employed to compare the midpoint potentials of truncated protein fragments of cytochrome *c*, cytochrome *c*_1_, Cu_A_, and cytochrome *c*_522_.^40^ Terminal oxidases (the membrane proteins cytochrome *c* oxidase and cytochrome *bd* oxidase) were also immobilised on the same modified gold nanoparticles and both non-turnover and catalytic signals were observed by cyclic voltammetry.^41–43^ Furthermore, immobilisation of cytochrome *bo*_3_ (another terminal oxidase) was compared on gold nanoparticles with different diameters, where smaller nanoparticles required lower overpotentials to reduce cytochrome *bo*_3_.^43^ The latter suggests that smaller particles have enhanced electronic coupling to the redox active sites of complex enzymes, presumably because smaller particles are able to come nearer the redox sites that are buried underneath a structured protein surface.

Instead of gold nanoparticles, it is possible to use redox-active proteins as conductive ‘interlayers’. For instance, we have demonstrated that a decaheme protein from *Shewanella oneindensis* MR-1, MtrC, could act as an electron relay between a porous indium tin oxide electrode and two redox enzymes (a hydrogenase and a fumarate reductase).^44^ Unlike metallic, conducting, nanoparticles, redox proteins have distinct redox states with discrete reduction potentials. Hence, it was observed that MtrC acted as an efficient diode regulating the direction of the enzymatic reactions. Interestingly, we observed that electron transfer to a fumarate reductase was more efficient when it was mediated by MtrC.

**Fig. 2:**
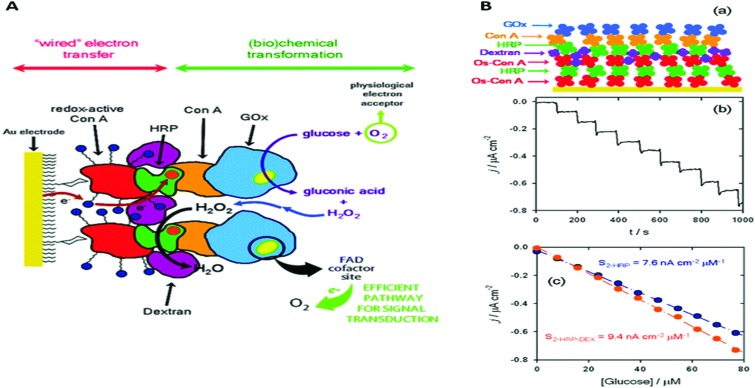
(A) Schematic representation of the glucose responsive supramolecular assembly that consists of redox-active ConA, horseradish peroxidase (HRP), ConA, dextran and glucose oxidase (GOx). (B-a) Simplified schematic representation for the data shown in this panel. (B-b) Chronoamperometry with increasing amounts of glucose. (B-c) Bioelectrocatalytic currents as a function of glucose concentration on this assembly with dextran (orange symbols) and without dextran (blue symbols). Reproduced as a composite from ref. 45 with permission from The Royal Society of Chemistry.

Recently, an approach was published were several of the approaches discussed so far were combined in a single system for glucose biosensing. In this system, the enzymatic reactions of glucose oxidation by glucose oxidase (GO) and reduction of hydrogen peroxide by horseradish peroxidase (HRP) were coupled together in a layer-by-layer assembly (Fig. 2A).^45^ Concanavalin A (ConA) a protein with carbohydrate binding sites, was used as an adhesive interlayer, where the interaction between ConA and the glycosylated GO and HRP was exploited for controlled layer-by-layer assembly. First, a recombinant version of ConA that carried an electroactive osmium-based tag was assembled on a modified gold electrode with mannose functional groups. The osmium modification of ConA (Os-ConA) made these protein particles redox active, thereby ‘wiring’ the layer of HRP that was immobilised on top. A layer of non-electroactive ConA was then formed on top to guide the subsequent assembly of a layer of GO. In the resulting assembly, glucose in the solution is oxidised by GO producing hydrogen peroxide, which diffuses to the layer containing HRP where it is reduced to water. Electrons required to reduce hydrogen peroxide were donated from the electrode to HRP *via* the redox-active Os-ConA layer. To prevent ‘cross talk’ between the GO layer and the osmium-modified ConA layer, a layer of dextran was deposited between the Os-ConA/HRP and ConA/GO layers. The addition of the dextran layer increased the efficiency of the glucose biosensor from 28% to 70%, while simultaneously increasing the sensitivity by 65% (Fig. 2B).^45^ The requirement of dextran illustrates an important limitation of many layer-by-layer assembled systems in that there is significant mixing between the layers.

A similar HRP–ConA–GO system approach was reported where the HRP layer was adsorbed on buckypaper (a carbon nanotube aggregate).^46^ The bucky paper was first modified with the redox compound 2,2′-azino-bis(3-ethylbenzothiazoline-6-sulphonic acid) or ABTS to enhance electron transfer to HRP. ConA was again used to bind a layer of GO. Both ABTS and ConA were modified with a pyrrole, which was electropolymerised to create the ABTS and ConA layers. A high current density was obtained of 1.1 ± 0.1 mA cm^–2^ at 0.1 V with 5 mM glucose and 64% of its initial current was retained after 15 days.^46^

### Multilayer assemblies in biophotoelectrochemistry

Semiconductive nanoparticles or quantum dots have also attracted interest in the field of biophotoelectrochemistry due to their ability to harvest energy from light. For instance, we have recently coupled dye-sensitized TiO_2_ nanocrystals to the decaheme MtrC using a layer-by-layer approach.^47^ By depositing MtrC prior to the dye-sensitized TiO_2_ nanocrystals, the MtrC protein layer separated the nanoparticles from the electrode and hence acted as an electron conduit. A photoswitching behaviour was observed that was dependent on the redox state of the electron mediator MtrC, hence confirming that photo-generated electrons were transferred from the dye-sensitized TiO_2_ nanocrystals to the underlying anode *via* MtrC.^47^

On a similar note, a light-controlled switch of electron transfer to cytochrome *c* in solution was demonstrated with CdSe/ZnS nanoparticles that were immobilised onto a gold electrode.^48^ Upon addition of cytochrome *c* to the solution, an enhancement of the photocurrent was observed in comparison to the generated photocurrent from only the immobilised CdSe/ZnS nanoparticles.^48^ The authors propose that the nanoparticles act as a barrier, preventing direct reduction of cytochrome *c* by the electrode. Upon illumination, cytochrome *c* was reduced by CdSe/ZnS nanoparticles, which in turn were reduced by the underlying electrode.

The Lisdat group has recently extended their layer-by-layer approach and reported on a multicomponent system for artificial photosynthesis that comprised of two different proteins, where DNA acted as a molecular ‘glue’ and cytochrome *c* as electron mediator to photosystem I (PS I).^49^ Layers of cytochrome *c* with PS I (at varying molar ratios) and DNA molecules were assembled layer-by-layer. They demonstrated an exponential increase of photocurrent up to 8 layers with no saturation at physiological pH.^49^ This scalable system has great biotechnological potential, although, as DNA is natively charged, the layer-by-layer approach requires the proteins to be positively charged (*i.e.* the formation of the assembly needs to be performed at pH conditions below the isoelectric point of the proteins). The system was shown to be stable, retaining 94% and 65% of the initial photocurrent after 9 days and 1 month of dry storage respectively.^49^

Redox polymers have also been employed in biophotoelectrochemistry. PSI and hydrogenase from *Desulfovibrio gigas* were electronically coupled with the use of two redox polymers: one with positive potential that could reduce PSI (with osmium complexes), and the other with negative potential (with either viologen or cobaltocene pendants) for the reduction of the hydrogenase.^50^ The viologen also actively scavenged oxygen which avoided oxidative de-activation of the hydrogenase.^50^

We have seen here examples of bioelectrochemical systems where layer-by-layer assembly was employed to either increase the enzyme loading on the electrode or to enhance or control electron exchange between the electrode and the redox proteins. In all cases, success of these systems depend on a sensitive interplay between diffusion of proteins or mediators within the layers and/or electron exchange between the proteins (electron hopping). Next, we will discuss supramolecular systems that have been specifically developed to provide a more native-like environment for redox enzyme complexes, in particular membrane bound enzymes.

## Lipid bilayer membrane based assemblies

Although bioelectrochemistry has proven to be exceptionally adapt in elucidating catalytic mechanisms of redox active enzymes, progress with membrane enzymes has been limited in comparison to globular ones. There are several reasons why progress has been hampered in this area. First, most electrode surfaces do not resemble a native-like environment for membrane enzymes. Hydrophobic domains of membrane enzymes will influence their interaction with the electrode and hence protein orientation and/or protein packing and by extension the electronic coupling to the electrode. Second, where large multi-subunit protein complexes are concerned, the quaternary structure can also be significantly disturbed by their interaction with the surface. Finally, in cases were membrane transport needs to be studied, the presence of a lipid membrane is key. The need of developing electrode assemblies that mimic the native membrane environment was apparent.

**Fig. 3:**
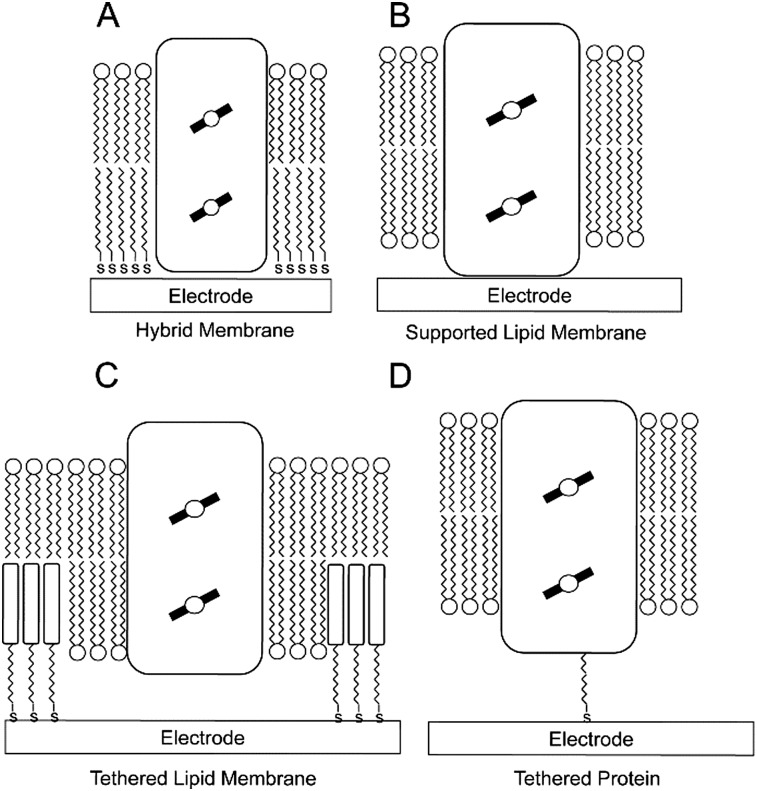
Schematic representations of the four basic membrane-modified electrodes in bioelectrochemistry. A redox-active integral membrane protein is graphically depicted as a rectangular box, with the redox-active cofactors as circles over filled black lines within the box: (A) a hybrid bilayer lipid membrane (hBLM); (B) a solid supported bilayer lipid membrane (sBLM); (C) a tethered bilayer lipid membrane (tBLM); and (D) a protein-tethered bilayer lipid membrane (ptBLM).

Attempts to develop electrode assemblies that included lipid–membrane-like structures emerged in the 1990s and were further characterized and optimized at the beginning of the 21st century. Early electrode assemblies formed bilayer lipid membranes on electrode surfaces that incorporated redox active membrane proteins while maintaining their electronic coupling to the electrode either *via* direct or mediated electron transport. These membrane-modified electrodes, which we have previously reviewed elsewhere,^51^ can be categorized into (a) hybrid bilayer lipid membrane (hBLM) system, (b) solid supported bilayer lipid membrane (sBLM) system, (c) tethered bilayer lipid membrane (tBLM) system, and (d) protein tethered bilayer lipid membrane (ptBLM) system (Fig. 3). In this section, we highlight recent advances that have been made with membrane-modified electrodes, with particular emphasis on supramolecular electrode assemblies where oligomeric enzyme complexes are studied or transmembrane charge transport is observed.

### Transmembrane transport

One advantage of membrane-modified electrodes is that both electron and transmembrane transport can be studied.

Photosynthesis and respiration rely on concerted reactions that couple electron transfer or redox reactions to proton or cation transfers across the membrane. Although cation transfer can in principle be detected with impedance spectroscopy,^52–57^ it can be difficult to deconvolute electron and cation transport in bioelectrochemical systems. Recently, several alternatives have been reported.

Proton transfer by the large multisubunit complex I of the mitochondrial respiratory system was studied by the group of De Lacey using a sBLM system (Fig. 3B).^58^ Complex I is a NADH:ubiquinone oxidoreductase and electron transfer to complex I was mediated by adding a naphthoquinone to the lipid membrane. Importantly, the sBLM was formed on a gold electrode modified with 4-aminothiophenol (4-ATP). The pH changes underneath the sBLM could be monitored using 4-ATP oxidation to aniline dimers as an electrochemical pH-probe. When complex I was activated by oxidising naphthoquinone electrochemically and adding NADH to the solution, the pH underneath the sBLM decreased by almost one unit. Similarly, the group of Hildebrandt recently measured the pH underneath a sBLM by determining the oxidation potential of decyl-ubiquinone (DUQ) in the sBLM, in this case with surface-enhanced infrared spectroscopy (SEIRA).^59^ The same DUQ was used to mediate electron transfer to an ubiquinol oxidase and proton pump, cytochrome *bo*_3_, incorporated in the sBLM assembly. Under turnover conditions, a shift in the oxidation potential of DUQ was apparent, indicating a pH increase of 0.8 units underneath the sBLM. In both cases (*i.e.*, complex I and cytochrome *bo*_3_), electron transfer to enzymes was mediated by membrane-embedded quinones and it can thus not be ascertained whether the pH changes underneath the sBLM are due to the proton pumping activity of the enzymes or due to proton release or uptake by the quinone upon oxidation or reduction by the electrode, respectively, or both.

**Fig. 4:**
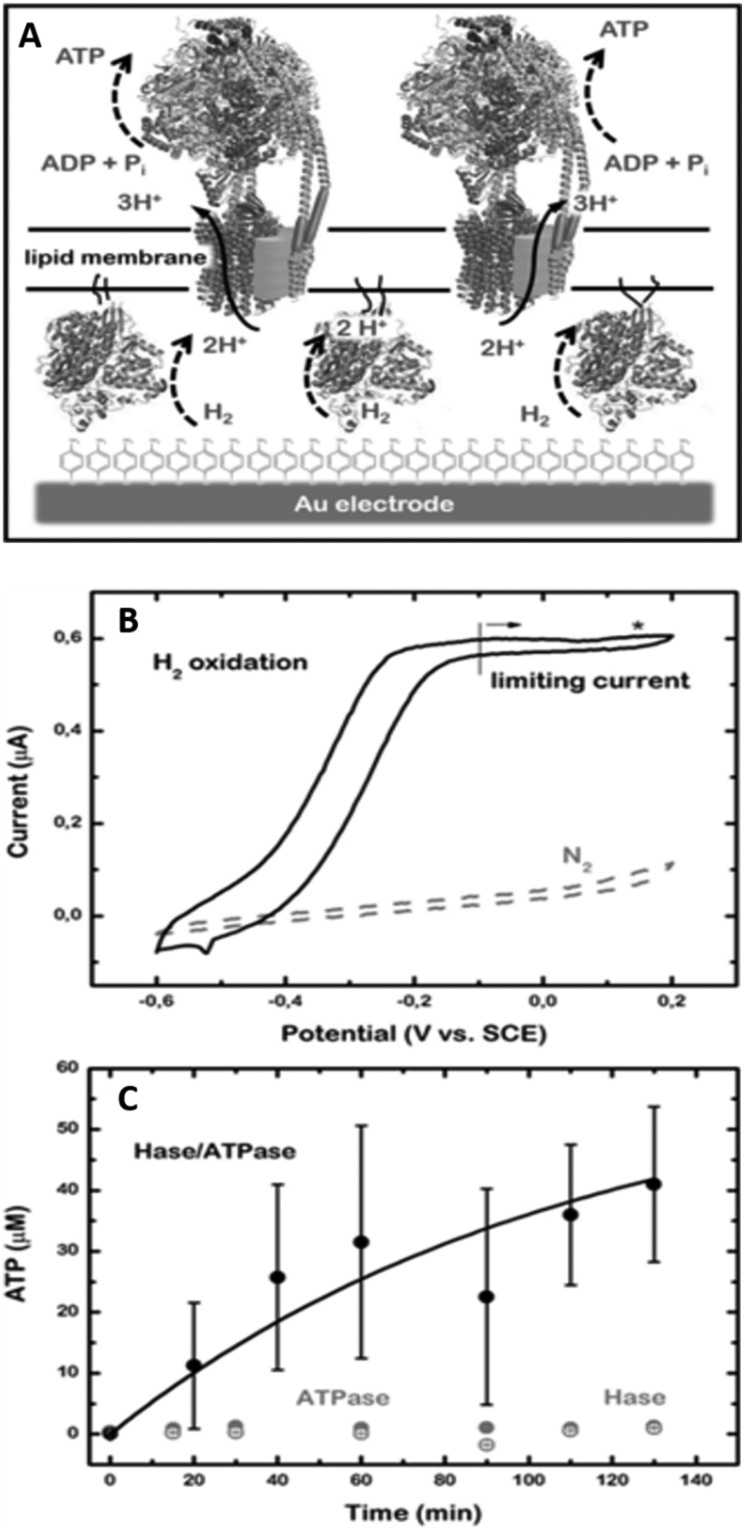
(A) Schematic representation of the [NiFeSe]-hydrogenase/F_1_F_0_-ATPase supramolecular ptBLM assembly on a gold electrode. (B) Cyclic voltammograms at a scan rate of 10 mV s^–1^ of the [NiFeSe]-hydrogenase/F_1_F_0_-ATPase ptBLM before (under N_2_) and after activation (under H_2_) of the [NiFeSe]-hydrogenase. (C) ATP production at 150 mV *vs.* SCE and 1 atm of H_2_ of a ptBLM that contained either both enzymes (black solid circles), only [NiFeSe]-hydrogenase (Hase; grey open circles), or only F_1_F_0_-ATPase (ATPase; grey solid circles). Reproduced as a composite from ref. 60.

Important in this respect is a recent work by the group of De Lacey,^60^ where a ptBLM (Fig. 3D) is formed on a 4-ATP SAM, but in this case embedding a [NiFeSe]-hydrogenase and a F_1_F_0_-ATPase (Fig. 4A). Here, electron transfer from [NiFeSe]-hydrogenase to the electrode is direct (Fig. 4B). H_2_ oxidation by hydrogenase generates protons and should thus reduce the pH underneath the ptBLM. The generated pH gradient is subsequently used to drive F_1_F_0_-ATPase and ATP is synthesised (Fig. 4C). This system requires the correct positioning of the two enzymes, which was achieved by the sequential, layer-by-layer, formation of the ptBLM. First, the [NiFeSe]-hydrogenase was covalently attached to the electrode. [NiFeSe]-hydrogenase contains a lipid tail that was then used to assemble a planar membrane from proteoliposomes containing F_1_F_0_-ATPase. The uniform orientation of [NiFeSe]-hydrogenase on the electrode was guided by electrostatic interactions, as well as by the dipole moment of the protein surface charge, allowing direct electron transport to the electrode.

This novel platform was compared to a previously established sBLM method where proteoliposomes with [NiFeSe]-hydrogenase were fused to form a planar lipid bilayer membrane on the electrode surface. In this system, direct exchange of electrons between [NiFeSe]-hydrogenase and the electrode was not observed, although mediated electron transport *via* methyl viologen confirmed the presence of [NiFeSe]-hydrogenase.^61^ The authors thus proposed that in this sBLM system, the [NiFeSe]-hydrogenase is located on top of the membrane (instead of sandwiched between the electrode and lipid membrane), thereby preventing direct electron transfer to the hydrogenase.^61^ Although the electron transfer between [NiFeSe]-hydrogenase and the electrode was mediated *via* methyl viologen, a 20-fold higher catalytic current was observed with sBLM system in comparison to a system with a soluble form of [NiFeSe]-hydrogenase, lacking its lipid tail,^61,62^ highlighting the importance of the membrane environment for the catalytic function of certain membrane enzymes.

Other studies have used membrane extracts rather than purified membrane enzymes. Membrane fractions of the yeast *Saccharomyces pombe* were capable of forming highly insulated membranes (>1 MΩ cm^2^) in a tBLM system (Fig. 3C).^63^ In this study, gramicidin ion channels were incorporated *in situ*, which decreased the resistance from 1.62 to 0.43 MΩ cm^2^. Cyclic voltammetry did not resolve a significant redox signal when ferricyanide was added, confirming that gramicidin formed ion channels and did not damaged the membrane (since gramicidin is not permeable to ferricyanide).^63^ The high resistance of this tBLM system could thus make it possible to study transmembrane charge transport, although this has not been fully exploited yet. Such approaches may be useful for biosensor technological applications where ion channels are employed as a tool to provide access to the underlying electrode. Such idea was further explored in a separate study where PorB class II porins were incorporated *in situ* and the transport of ferricyanide ions was electrochemically characterised.^64^

Thylakoid membranes from spinach, packing PSI, PSII, chlorophyll, and other pigments associated with photosynthesis, were immobilised on electrodes for their use as a bioanode for water oxidation in a two-chamber biosolar cell.^65^ Conjugated oligoelectrolytes (CEO), which are conductive but not redox active, were systematically tested for their ability to boost photocurrents and the best performing CEO increased the current 2.3-fold.^65^

### Membrane protein supercomplexes

In a recent example of a tBLM approach (Fig. 3C), we have studied the catalytic properties of an oxygen tolerant [NiFe]-hydrogenase from *Ralstonia eutropha*.^66,67^ One of the aim of these studies was to test whether higher oligomeric complexes of [NiFe]-hydrogenase in the membrane would influence oxygen tolerance of this membrane-bound hydrogenase; previous studies on the [NiFe]-hydrogenase from *Ralstonia eutropha* were all performed on water soluble sub-complex in which a transmembrane subunit was removed.^68^ tBLM systems were assembled using native membrane fractions from *Ralstonia eutropha* that overexpressed the [NiFe]-hydrogenase. Membrane extracts rather than purified enzymes were used because the full heterotrimeric membrane-bound form of this enzymes tends to dissociate upon purification. Furthermore, the use of membrane extracts ensured that the catalytic mechanism was studied in a near-native environment. Electron transport from the [NiFe]-hydrogenase was mediated by ubiquinone or menaquinone (the enzyme's native substrates) in the tBLM. Catalytic activities obtained with the tBLM system were maintained under aerobic conditions and highly oxidative potentials,^66^ in contrast to previous studies with a water-soluble subcomplex of the enzyme.^68^ At the time, we ascribed the enhanced tolerance to oxygen to the oligomeric state of hydrogenase in the lipid membrane. However, a more recent study by the Armstrong group showed that the water-soluble subcomplex of such a [NiFe]-hydrogenase also depends on oligomerisation and hence this does not seem to be a feature specific to the membrane environment.^69^

**Fig. 5:**
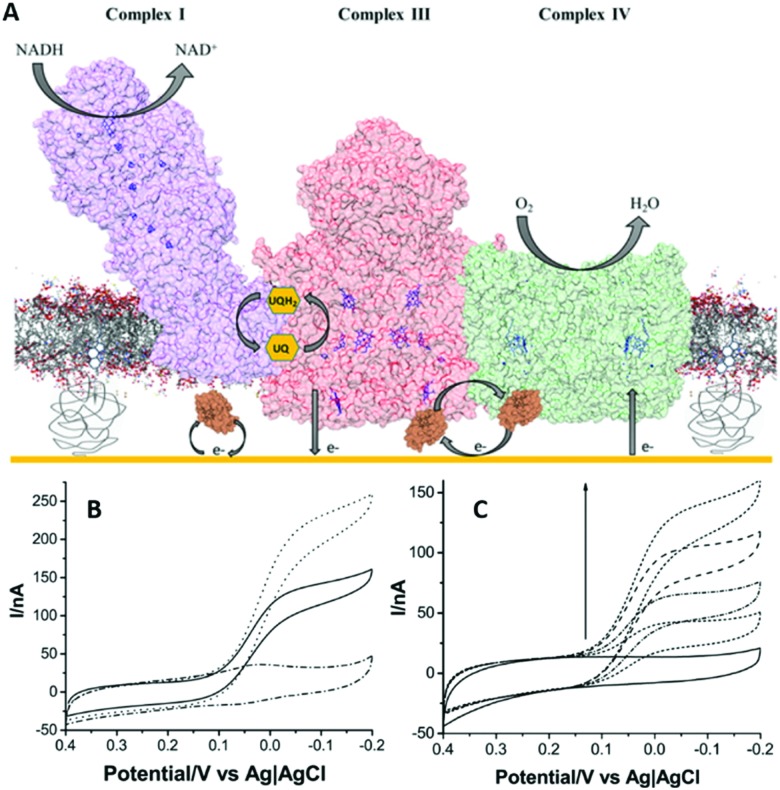
(A) Schematic representation of the electron transport chain metabolon incorporated in a tethered lipid bilayer membrane on a gold electrode. (B) Cyclic voltammograms at a scan rate of 5 mV s^–1^ of the metabolon with oxidised cytochrome *c* (solid line); complex IV electrodes with reduced cytochrome *c* (dotted line); and only cytochrome *c* containing control bilayers (dash-dotted line). (C) Cyclic voltammograms at a scan rate of 5 mV s^–1^ of the metabolon with increasing amounts of oxidised cytochrome *c* (0–5 μM). Reproduced as a composite with permission from ref. 70. Copyright 2016 American Chemical Society.

Although highly insulating lipid bilayer membranes are needed to study ion translocation, low-resistive tBLM systems have benefits when electrocatalysis is studied and transfer of ions, metabolites or small proteins across the membrane is required. A recent report took advantage of a low-resistive tBLM to construct a mimetic of the entire mitochondrial inner membrane on a gold electrode, aiming to study functional aspects of the supercomplex between complexes I, III and IV (Fig. 5).^70^ The gold electrode was modified with a mixed monolayer of β-mercaptoethanol and cysteamine, where the amine functional groups were required to couple PEGylated cholesterols *via* the *N*-hydroxysuccinimide. The cholesterol in turn was used to create a tBLM system (Fig. 3C).^70^ Complexes I, III, and IV were purified and reconstituted in liposomes containing ubiquinone, which they were used to form the tBLM. Cytochrome *c* was added in solution to complete the mitochondrial electron-transport chain and the tBLM defects allowed diffusion of cytochrome *c* to the electrode. This was illustrated by a tBLM formed with liposomes that do not contain any protein, where linear non-turnover signals were observed when up to 5 μM of cytochrome *c* was added.^70^

Insights into the electron transport chain were obtained from this system, drawing on the ability to study the whole electron transport chain with bioelectrochemistry and to depict the contributions of each protein in this process.

### Novel membrane-modified surfaces

A new approach for anchoring lipid membranes has emerged that addresses the limitations of the hBLM and sBLM systems with regards to the incorporation of integral membrane proteins. In hBLM, tBLM, and sBLM systems, the limited space between the electrode and the lipid bilayer membrane can hinder the lateral diffusion of the protein, or even lead to protein degradation due to intermolecular interactions between the protein and the solid support. To this end, Zr^4+^ ions were employed as an ionic superglue in forming a water cushioned lipid bilayer membrane on a modified gold electrode with phosphate groups.^71^ Employing coordination chemistry, Zr^4+^ ions act as linkage between the phosphate functional groups on the electrode and the phospholipid headgroups of the electrode-facing lipid bilayer membrane. Neutron reflectivity measurements revealed a hydrated layer with 47 Å thickness between the SAM and the lipid bilayer membrane.^71^

Another similar approach has also emerged where a floating supported lipid bilayer membrane was constructed on a ω-thiolipid modified gold electrode.^72^ In this system, neutron reflectivity measurements revealed a water layer with 17 Å thickness, which was decreased with increased chain length of the bilayer lipids,^72^ providing experimental control of the thickness of the water layer.

A relatively easy to implement approach for generating multilayers of lipid bilayers was shown with the use of poly l-lysine.^73^ This layer-by-layer membrane system could expand the protein multilayer advantages to membrane proteins in a near native environment for bioelectrochemistry, although the electronic coupling of those proteins in this system has not been shown yet. Similarly, both the Zr^4+^ anchored water-cushioned- and the floating supported lipid bilayer membrane approaches have not yet been employed in the electrochemical interrogation of membrane proteins, but they show potential.

## Conclusions and outlook

PFE has undoubtedly paved our understanding of inter- and intra-protein electron transfer and, by extension, of metalloenzymes in biology. However, a big number of metalloenzymes are integral or associated membrane proteins, which has inspired the development of electrode architectures that resemble a membranous environment. Moreover, electrochemistry has found great interest in the field of biosensor and fuel cell technologies, further emphasizing its versatility in addressing both research and biotechnological needs. In this featured article, we have looked at multilayer protein assemblies in layer-by-layer films, aided by non-conductive, conductive, or semiconductive nanoparticles; and how these very recently have been combined towards artificial photosynthesis and biosensor technologies. We have also discussed recent advances in the bioelectrochemistry of membrane proteins with a focus on the new lipid bilayer electrode architectures that are emerging. It is envisaged that electrochemical methods will expand to ion channels, and more generally to membrane transport processes. On the other hand, proteins pose several limitations that are inherent in their design when they are employed for technological applications. To this end, protein engineering holds great potential for the production of purpose-specific enzymes.
